# Restoration of peripheral ILC3s by washed microbiota transplantation improves lipid profiles in hyperlipidemia patients

**DOI:** 10.3389/fimmu.2025.1688070

**Published:** 2025-12-03

**Authors:** Yao-Fei Wei, Yi-Shu Wang, Jia-Yin Song, Hao Wang, Xing-Xiang He, Hao-Jie Zhong

**Affiliations:** 1 Department of Gastroenterology, Research Center for Engineering Techniques of Microbiota-Targeted Therapies of Guangdong Province, The First Affiliated Hospital of Guangdong Pharmaceutical University, Guangzhou, China; 2 Nanfang Hospital, Southern Medical University, Guangzhou, China; 3 Guangdong Provincial Key Laboratory for Research and Evaluation of Pharmaceutical Preparations, Guangdong Pharmaceutical University, Guangzhou, China; 4 Department of Rheumatology, Shenzhen Traditional Chinese Medicine Hospital, The Fourth Clinical Medical College of Guangzhou University of Chinese Medicine, Shenzhen, China

**Keywords:** hyperlipidemia, group 3 innate lymphoid cells, washed microbiota transplantation, lipid metabolism, immunometabolism

## Abstract

**Background:**

The contribution of circulating group 3 innate lymphoid cells (ILC3s) to lipid dysregulation has remained poorly defined, and the mechanisms through which washed microbiota transplantation (WMT) improves lipid metabolism require further clarification.

**Methods:**

Peripheral ILC subsets and plasma IL-22 were assessed in hyperlipidemia patients and healthy controls. The lipid-lowering effects of WMT were evaluated in a prospective cohort without lipid-lowering medications. Gut microbial and plasma metabolite profiles before and after WMT were analyzed. A hyperlipidemic mouse model was used to determine whether healthy microbiota promote hepatic ILC3 homing via integrin α4.

**Results:**

Hyperlipidemia was characterized by reduced circulating ILC3s, integrin α4^+^ ILC3s, and plasma IL-22, all of which showed inverse correlations with TC, TG, LDL-C, non-HDL-C, and ApoB. Significant lipid improvements were achieved after WMT, accompanied by increased circulating ILC3s and integrin α4^+^ ILC3s, paralleling reductions in TC and LDL-C. WMT induced marked remodeling of gut microbiota and plasma metabolites, including taxa and metabolites positively associated with ILC3 restoration and lipid improvement. In hyperlipidemic mice, healthy microbiota transplantation increased hepatic ILC3 and integrin α4^+^ ILC3 accumulation and improved lipid levels, whereas integrin α4 blockade impaired ILC3 liver homing and attenuated the metabolic benefit.

**Conclusion:**

Hyperlipidemia is associated with depletion of circulating ILC3s and reduced IL-22. Restoration of ILC3 subsets and enhancement of integrin α4–dependent hepatic homing are achieved after WMT, accompanying improvements in lipid metabolism.

## Graphical Abstract

**Figure:**
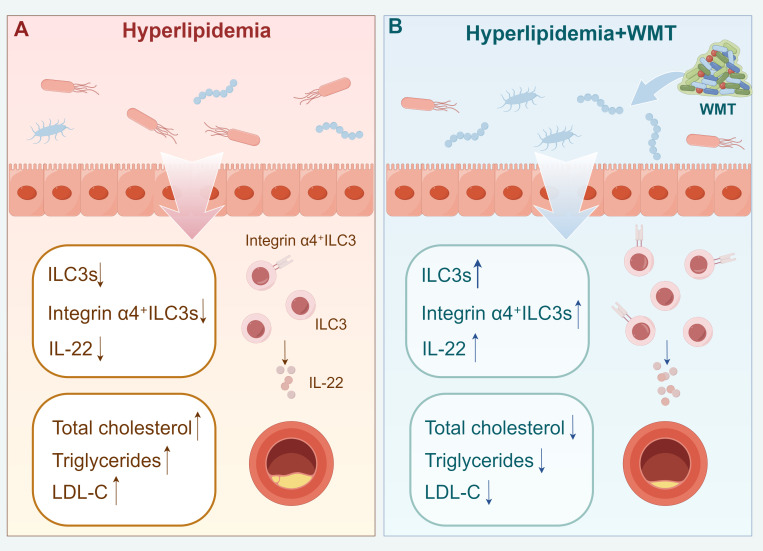
The graphical abstract illustrates the immunometabolic mechanism by which washed microbiota transplantation (WMT) improves hyperlipidemia. Hyperlipidemia is characterized by reduced circulating ILC3s, decreased integrin α4⁺ ILC3s, and diminished IL-22, accompanied by elevated lipid levels. Following WMT, gut microbial composition and metabolites are remodeled, leading to restoration of circulating ILC3s, increased integrin α4 expression, and enhanced hepatic homing of ILC3s. These immune alterations contribute to improved lipid metabolism, highlighting a microbiota–ILC3–IL-22 axis that mediates the therapeutic benefit of WMT.

## 1 Introduction

Hyperlipidemia affects more than 40% of Chinese adults and is a major contributor to cardiovascular morbidity and mortality (1–3). Although lipid-lowering agents such as statins are widely prescribed, many patients fail to achieve target lipid levels due to limited efficacy, adverse effects, or poor treatment adherence (4). These limitations highlight the need for safe and effective alternative strategies to control dyslipidemia and reduce long-term health risks.

Mounting evidence indicates that the gut microbiota plays a pivotal role in lipid metabolism. Patients with dyslipidemia frequently exhibit gut microbial dysbiosis (5, 6), and fecal microbiota transplantation (FMT) from normolipidemic donors has been shown to ameliorate lipid abnormalities in animal models (7). Our previous studies demonstrated that washed microbiota transplantation (WMT)—a refined FMT approach involving standardized washing to remove fecal impurities—significantly improved lipid profiles in hyperlipidemia patients without altering concurrent medication regimens (8, 9). However, the efficacy of WMT as a standalone intervention and the underlying mechanistic pathways remain to be elucidated.

Group 3 innate lymphoid cells (ILC3s), predominantly located at mucosal sites, are key mediators of intestinal barrier integrity (10). Beyond their mucosal functions, ILC3s contribute to systemic metabolic regulation, notably through secretion of interleukin-22 (IL-22) and modulation of dendritic cell activity (11–13). Importantly, the gut microbiota exerts a profound influence on ILC3 abundance and function. Preclinical studies suggest that microbiota-based interventions, including FMT, can ameliorate intestinal injury and metabolic dysfunction via ILC3 modulation (14–16). Despite these insights, the phenotypic characteristics of circulating ILC3s in hyperlipidemia, as well as their potential contribution to systemic lipid dysregulation, have not been elucidated. Moreover, it remains unclear whether WMT can modulate these immune subsets in humans to improve lipid metabolism.

To address these gaps, we comprehensively profiled circulating ILC subsets and plasma IL-22 in hyperlipidemia patients and healthy controls, evaluated the lipid-lowering efficacy of WMT in a prospective cohort without lipid-lowering medications, and performed microbiome and metabolomic analyses to characterize treatment-associated changes. Furthermore, using a hyperlipidemic mouse model, we investigated whether donor microbiota promote hepatic ILC3 homing through integrin α4—a critical trafficking molecule implicated in gut–liver immune communication. Collectively, this study delineates an immunometabolic axis linking the gut microbiota, ILC3s, and lipid homeostasis, and provides mechanistic insight into the therapeutic potential of WMT for dyslipidemia.

## 2 Methods

### 2.1 Blood samples and data collection

Peripheral blood samples were collected from patients with hyperlipidemia and healthy controls between September 2020 and December 2024 at the First Affiliated Hospital of Guangdong Pharmaceutical University. All blood samples for lipid profile measurement were obtained in the morning after an overnight fast of at least 8 hours. Patients with hyperlipidemia who had comorbid malignancies, autoimmune diseases, or infectious diseases were excluded from the study. Demographic and clinical data, including gender, age, body mass index (BMI), triglycerides (TG), total cholesterol (TC), high-density lipoprotein cholesterol (HDL-C), low-density lipoprotein cholesterol (LDL-C), non-high-density lipoprotein cholesterol (non-HDL-C), and apolipoprotein B (ApoB), were collected from patients with hyperlipidemia. Hyperlipidemia was diagnosed according to the following criteria: total cholesterol ≥ 6.20 mmol/L, LDL-C ≥ 4.10 mmol/L, triglycerides ≥ 2.30 mmol/L (9). The study protocol was approved by the Ethics Committee of the First Affiliated Hospital of Guangdong Pharmaceutical University (Approval No. 2021146), and written informed consent was obtained from all participants.

### 2.2 Animal experiments

Eighteen 10-week-old male C57BL/6 mice were randomly assigned to three groups. After one week of acclimatization, all mice were fed a high-fat, high-cholesterol diet and provided with 30% sucrose water for four weeks to induce hyperlipidemia. During model induction, one group received daily oral gavage of microbiota derived from hyperlipidemic mice, another group received daily gavage of microbiota from healthy mice, and the third group received daily gavage of healthy-donor microbiota together with a weekly intraperitoneal injection of 50 μg integrin α4–neutralizing antibody (RM4995, Biodragon). All animal procedures were approved by the Animal Ethics Committee of the First Affiliated Hospital of Guangdong Pharmaceutical University.

Fecal samples from healthy and hyperlipidemic mice were collected into sterile Eppendorf tubes. Each sample (0.1 g) was suspended in 1 mL of normal saline for 10 minutes, homogenized, and centrifuged at 1,000 rpm for 3 minutes. The resulting suspension was centrifuged again at 3000 rpm for 5 minutes, and the pellet was washed twice with normal saline. After discarding the supernatant, 1 mL of saline was added to the microbiota pellet to prepare the bacterial suspension, which was administered by oral gavage daily for four weeks.

At the end of the treatment period, liver tissues were collected for flow cytometric analysis, and plasma samples were obtained for lipid measurements.

### 2.3 Flow cytometry

For human samples, peripheral blood mononuclear cells (PBMCs) were obtained through red blood cell lysis using a buffer solution (Solarbio, Beijing). For surface marker identification, cells were stained with human monoclonal antibodies against lineage markers, including CD3 (HIT3a), CD5 (UCHT2), CD11c (3.9), CD16 (B73.1), CD19 (HIB19), and TCRαβ (IP26), as well as CD45 (H130), CD127 (A019D5), CD117 (A3C6E2), CD297 (BM16), and integrin α4 (9F10) (all purchased from BioLegend). Non-viable cells were excluded by staining with 7-aminoactinomycin D (7-AAD) viability dye (BioLegend). To assess the IL-22–producing capacity of ILC3s, PBMCs were first stained for surface markers and then stimulated with PMA/ionomycin (BioLegend) in the presence of GolgiPlug protein transport inhibitor (BD) for 4 hours. Subsequently, intracellular cytokine staining was performed using the Foxp3/Transcription Factor Staining Buffer Set (eBioscience) according to the manufacturer’s instructions, together with an anti–IL-22 antibody (2G12A41; BioLegend). Total ILCs were defined as 7-AAD^-^CD45^+^lineage (CD3, CD5, CD11c, CD16, CD19, and TCRαβ)^-^CD127^+^ lymphocytes. ILC1s were identified as CD117^-^CD294^-^, ILC2s as CD294^+^, and ILC3s as CD117^+^CD294^-^ (17).

For mouse liver samples, hepatic lymphocytes were isolated by mechanically dissociating liver tissue through a 70-µm strainer in PBS. The resulting suspension was centrifuged at 1,500 rpm for 5 minutes at 4 °C, and the pellet was resuspended in 40% Percoll. This suspension was layered onto 100% Percoll and centrifuged at 800 × g for 20 minutes without acceleration or braking to enrich lymphocytes. The interphase was collected, washed, and prepared for flow cytometry. Mouse ILC3s were characterized using antibodies against CD3ϵ, CD19, CD11c, CD11b, CD5, TER-119, CD45, CD127, integrin α4, IL-22 (BioLegend), and RORγt (eBioscience). Intracellular staining was performed using the Foxp3 staining kit (eBioscience) following the manufacturer’s instructions. All samples were acquired on a CytoFLEX flow cytometer (Beckman Coulter), and data were analyzed using CytoExpert software version 2.3.

### 2.4 Enzyme-linked immunosorbent assay

Plasma levels of human IL-22 were quantified using ELISA kits (BioLegend, 434504), following the manufacturer’s instructions and established protocols.

### 2.5 Patients with hyperlipidemia treated with WMT

Patients with hyperlipidemia who received WMT at the First Affiliated Hospital of Guangdong Pharmaceutical University between April 2017 and December 2024 were enrolled in this study. Patients were excluded if they had used lipid-lowering agents or corticosteroids, had taken antibiotics within the past month, or had missing key clinical or follow-up data. In addition, sex- and age-matched hyperlipidemia patients who received lifestyle intervention alone or lipid-lowering medications alone during the same period were included as control groups to compare the therapeutic effects of the three treatment strategies. The study protocol was approved by the Ethics Committee of the First Affiliated Hospital of Guangdong Pharmaceutical University (Approval No. 201798), and written informed consent was obtained from all participants.

### 2.6 WMT procedure

Healthy donors were selected based on comprehensive screening procedures, including questionnaires, physical examinations, blood analyses, and stool tests (18). The preparation of the washed microbiota suspension followed standardized protocols described in prior studies (19). Specifically, 100 g of fresh fecal material were homogenized in 500 mL of saline and then subjected to microfiltration to remove particles ≥19 μm using an automated purification system (FMT Medical, Nanjing, China) to isolate the microbial fraction. The bacterial pellet was obtained by centrifugation at 1100×g for 3 min and washed three times with saline. The resulting microbial paste was weighed, and 10 g of the pellet were resuspended in 20 mL of saline to prepare the final washed microbiota suspension. The interval from donor stool collection to microbiota transplantation was strictly controlled within 2 hours to ensure microbial viability and consistency.

WMT was administered by delivering 120 mL of fecal microbiota suspension daily for three consecutive days, either through a nasojejunal tube (middle gastrointestinal route) or a transendoscopic enteral tube (lower gastrointestinal route), depending on each patient’s clinical condition and personal preference. This three-day infusion protocol constituted a single treatment course, with subsequent courses repeated at one-month intervals. Patients did not receive antibiotic pretreatment before WMT. Donor microbiota preparations were randomly assigned to recipients.

### 2.7 Clinical data collection

Clinical information was extracted from electronic medical records and included demographic characteristics, BMI, smoking and alcohol consumption status, history of hypertension and type 2 diabetes mellitus, current medication use, indications for WMT, route of WMT administration (lower or mid-gastrointestinal tract), WMT-related adverse events, and laboratory parameters, including triglycerides, total cholesterol, HDL-C, and LDL-C.

### 2.8 16S rRNA gene sequencing, library preparation, and bioinformatic analysis

Fecal samples collected before and after treatment were obtained using fecal DNA preservation tubes (Invitek, Germany) and stored at –80 °C until further processing. Genomic DNA was extracted using the CTAB method and diluted to 1 ng/µL after quality assessment on 1% agarose gels. The V3–V4 region of the 16S rRNA gene was amplified using primers 341F (5’-CCTAYGGGRBGCASCAG-3’) and 806R (5’-GGACTACNNGGGTATCTAAT-3’). PCR was performed with Phusion High-Fidelity PCR Master Mix under standard cycling conditions. Amplicons were checked on 2% agarose gels, pooled in equimolar amounts, purified (TianGen), and used to construct sequencing libraries with the NEBNext Ultra DNA Library Prep Kit. Libraries were quality-checked on an Agilent 5400 system and sequenced on the Illumina platform to generate 250 bp paired-end reads.

Bioinformatic processing followed the QIIME2 “Atacama soil microbiome” workflow. Raw FASTQ files were imported, quality-filtered, denoised, merged, and chimera-removed using DADA2 to obtain ASVs. Taxonomy was assigned using a GREENGENES 13_8 99% classifier trimmed to the V3–V4 region, and mitochondrial/chloroplast reads were removed. Alpha diversity and beta diversity were calculated and visualized using PCoA. Differentially abundant taxa were identified using ANCOM, ANOVA, Kruskal–Wallis, or LEfSe. Co-occurrence networks were generated using Spearman correlations. Default parameters were applied unless otherwise stated. Microbial sequencing data were deposited in the Sequence Read Archive database under PRJNA number 1364992.

### 2.9 Metabolite extraction, UHPLC–MS/MS analysis, and data processing

Whole blood samples collected before and after WMT were centrifuged at 3,000 rpm for 5 minutes to obtain plasma, which was then stored at –80 °C until analysis. Plasma (100 μL) were mixed with pre-chilled 80% methanol, vortexed, incubated on ice for 5 min, and centrifuged at 15,000 g for 20 min at 4 °C. The supernatant was diluted to 53% methanol, transferred to a fresh tube, centrifuged again, and the final supernatant was used for LC–MS/MS analysis.

UHPLC–MS/MS was performed on a Vanquish UHPLC system coupled to an Orbitrap Q Exactive™ HF/HF-X mass spectrometer (Thermo Fisher). Samples were injected onto a Hypersil Gold column (100 × 2.1 mm, 1.9 μm) using a 12-min gradient at 0.2 mL/min. Eluent A was 0.1% formic acid in water, and eluent B was methanol. Data were acquired in both positive and negative ion modes under standard instrument settings.

Raw UHPLC–MS/MS data were processed using XCMS for peak picking, alignment, and quantification. Metabolites were identified by matching adduct ions (± 10 ppm) to an in-house spectral database. After removing background ions, quantitative values were normalized to relative peak areas. Metabolites with a coefficient of variation >30% in QC samples were excluded. Data processing was performed in Linux (CentOS 6.6) using R and Python.

### 2.10 Measurement of plasma lipid levels in mice

Plasma lipid profiles in mice were measured using an automated biochemical analyzer (Chemray 800). Assessed parameters included TC, TG, and LDL, following the manufacturer’s operating procedures.

### 2.11 Statistical analysis

Statistical analyses were conducted using SPSS (version 22.0; IBM, Armonk, NY, USA) and GraphPad Prism (version 8; GraphPad, San Diego, CA, USA). Sample size estimation was performed based on preliminary experimental results using the online Power and Sample Size Calculators, ensuring adequate statistical power for primary outcomes. Continuous variables are presented as mean ± standard deviation or median (interquartile range), depending on data distribution. Categorical variables are expressed as frequencies and percentages. Between-group comparisons were performed using the Student’s t-test or Wilcoxon rank-sum test for continuous data, and the chi-square test or Fisher’s exact test for categorical data. Correlations between two continuous variables were assessed using Pearson or Spearman correlation analysis. Multivariable linear regression was employed to adjust for potential confounding effects on peripheral blood ILC3 levels and WMT efficacy. A two-tailed P-value <0.05 was considered statistically significant.

## 3 Results

### 3.1 WMT is a safe and effective strategy for lipid reduction in hyperlipidemia

**Figure 1:**
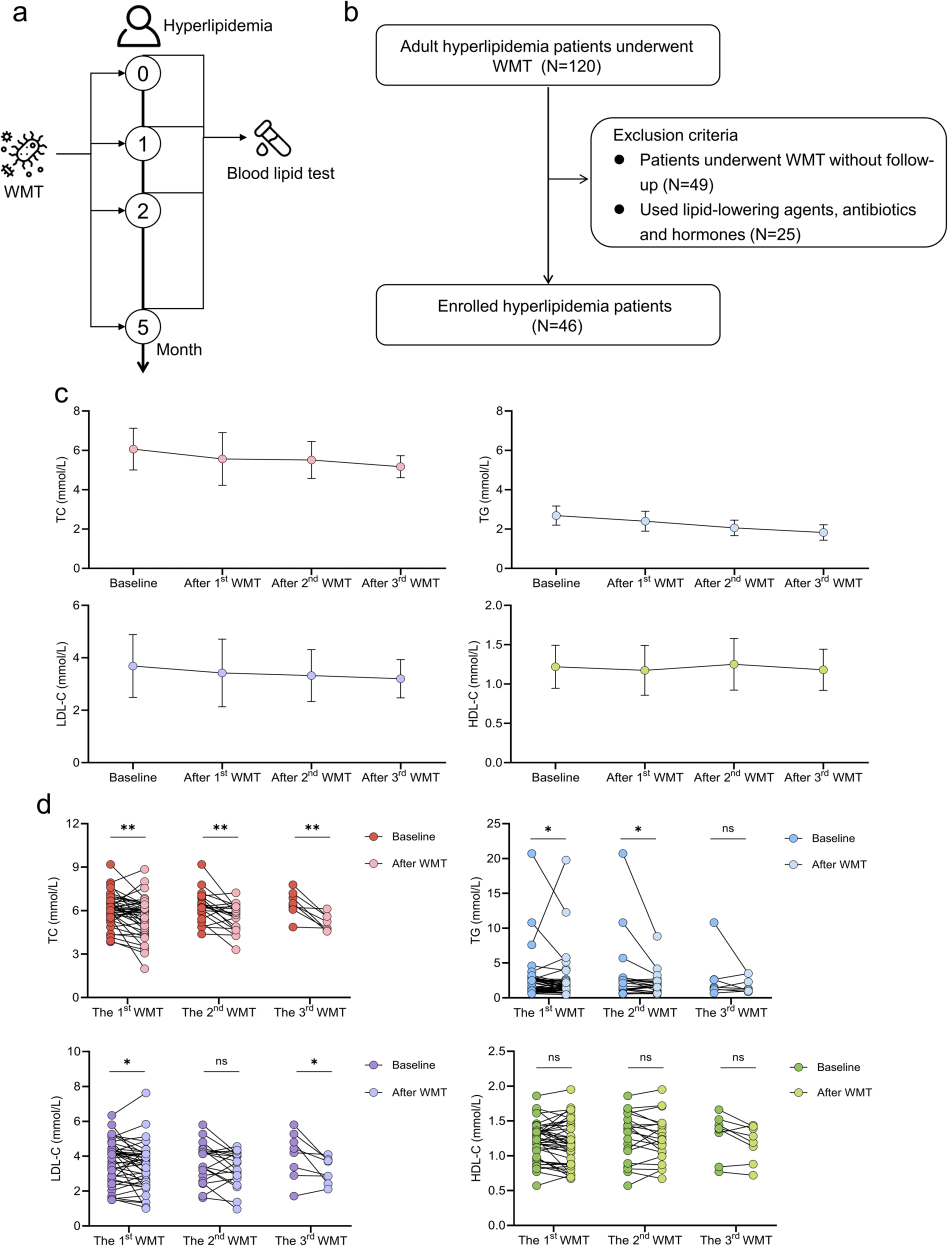
Effects of WMT on lipid profiles in patients with hyperlipidemia **(a)** Study design. **(b)** Flowchart of the study population. **(c)** Longitudinal changes in lipid profiles following WMT. **(d)** Comparison of lipid profiles before and after WMT. Data are presented as mean ± standard error. *p < 0.05; **p < 0.01; ns, not significant.

We first evaluated the clinical efficacy of WMT in hyperlipidemia by analyzing lipid profile changes in patients who underwent the procedure without concurrent lipid-lowering therapy. A total of 46 patients with hyperlipidemia met the predefined criteria and were included (**Figures 1a, b**). Baseline characteristics and indications for WMT are summarized in **Supplementary Tables S1** and **S2**. All patients completed the first WMT course, 27 completed two courses, and 10 completed three courses. The median interval between the first and second courses was 35.50 (33.25–45.75) days, between the second and third courses was 43.00 (34.00–63.00) days, and between the third course and follow-up was 112.50 (89.00–243.00) days.

**Figure 1c** shows a progressive decline in TC, TG, and LDL-C levels in patients with hyperlipidemia following multiple courses of WMT. Even after a single WMT course, TC, TG, and LDL-C were significantly reduced compared to baseline. After two courses, TC and TG remained significantly decreased, while after three courses, TC and LDL-C were further reduced (**Figure 1d**). In addition to lipid profile improvements, BMI and fasting insulin levels were significantly reduced after WMT (**Supplementary Figure S1**), suggesting broader metabolic benefits of the intervention. Although the reduction in TC achieved by WMT was less pronounced than that typically observed with lipid-lowering medications, it was substantially greater than that achieved through lifestyle intervention alone (**Supplementary Figure S2**), indicating that the therapeutic effect of WMT is independent and not merely due to behavioral changes or spontaneous fluctuations in lipid levels.

To clarify the influence of clinical characteristics on WMT efficacy, we conducted multivariable linear regression analyses including sex, age, overweight status (BMI≥25), alcoholism, smoking history, presence of fatty liver, number of WMT cycles, and route of administration. The results showed that patients with alcoholism experienced greater reductions in total cholesterol after WMT (β=-0.77, p=0.036); those with alcoholism (β=-3.18, p<0.001), those receiving more treatment cycles (β=-0.73, p=0.004), or those treated via the lower gastrointestinal route (β=-1.36, p=0.027) exhibited larger decreases in triglycerides; and older patients (β=-0.02, p=0.046) showed more pronounced reductions in LDL-C. These findings suggest that patients with these characteristics may obtain greater benefit from WMT.

Across 83 WMT procedures, only one mild adverse event (fatigue) was reported within one week after treatment, and it resolved spontaneously. The overall adverse event rate was 1.20%, with no serious complications observed. Sixteen patients were followed up three months after treatment, and no long-term adverse reactions were detected. Among them, three patients had abnormal peripheral immune cell counts (lymphocytes, neutrophils, monocytes, eosinophils, or basophils) before treatment; one of these patients returned to normal levels at the 3-month follow-up. The remaining 13 patients, all of whom had normal immune cell profiles before WMT, showed no abnormalities during follow-up. These findings indicate that WMT is a safe and effective approach for improving lipid profiles in patients with hyperlipidemia; however, studies with larger sample sizes and longer follow-up durations are still needed to further confirm the safety profile of WMT.

### 3.2 Peripheral ILC3 proportions are decreased in hyperlipidemia and inversely correlate with disease severity

**Figure 2:**
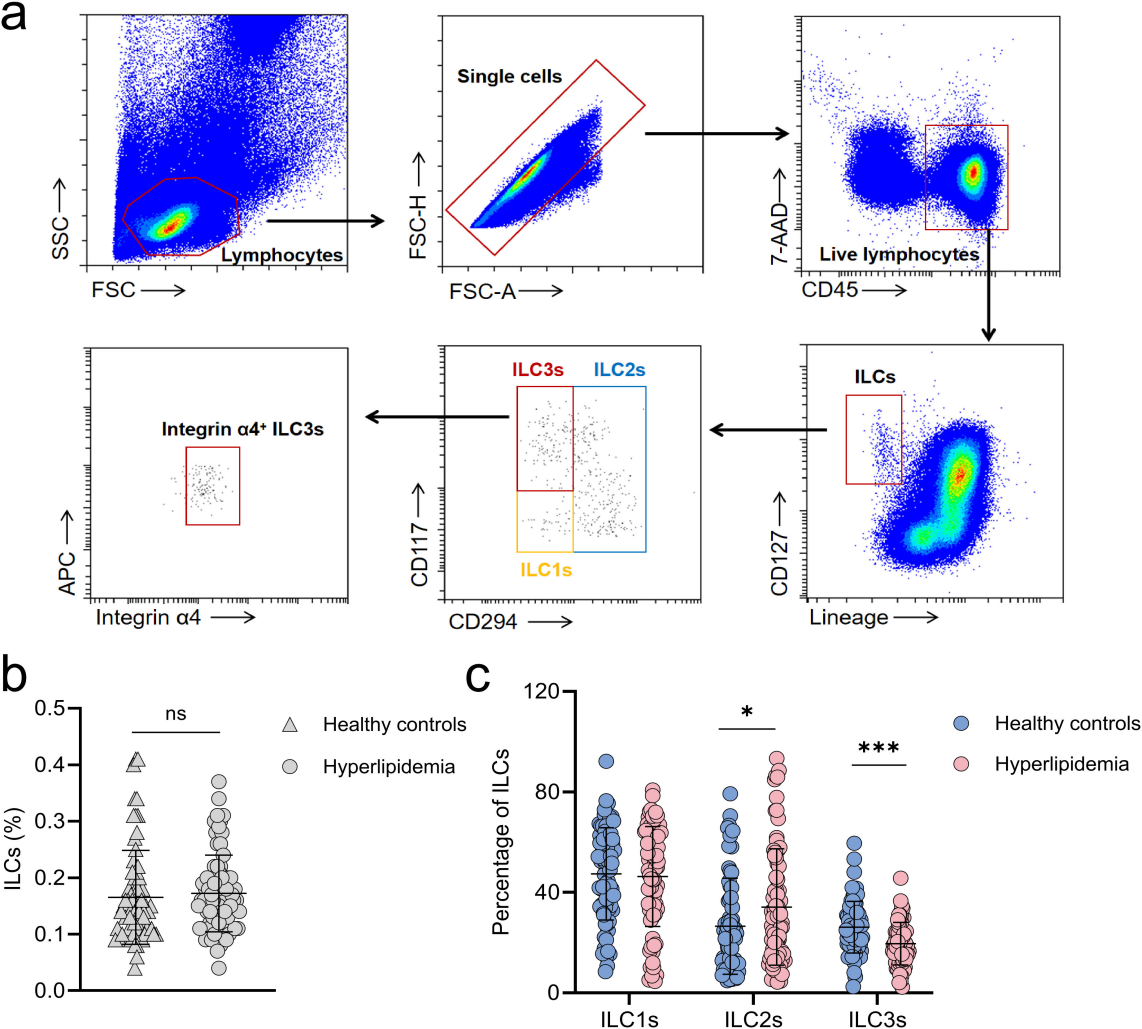
Circulating ILC3 levels are reduced in patients with hyperlipidemia. **(a)** Gating strategy for identifying circulating ILCs in healthy controls (n=72) and patients with hyperlipidemia (n=75). **(b)** Comparison of total circulating ILCs between the two groups. **(c)** Comparison of ILC subsets (ILC1s, ILC2s, and ILC3s) between healthy controls and hyperlipidemia patients. FSC, forward scatter; SSC, side scatter; ILCs, innate lymphoid cells; ILC1s, group 1 ILCs; ILC2s, group 2 ILCs; ILC3s, group 3 ILCs. Lineage = CD3, CD19, CD16, CD11c, CD11b, CD5, and TCRαβ. *p < 0.05; ***p < 0.001; ns, not significant.

Although gut microbiota are known to regulate host lipid metabolism through ILC3s (16), their role in hyperlipidemia remains unclear. To investigate this, peripheral blood samples from 72 healthy controls and 75 hyperlipidemia patients were analyzed by flow cytometry. There were no significant differences in age or sex between the two groups (**Table 1**). The gating strategy for identifying ILC subsets is illustrated in **Figure 2a**.

**Table 1:** Characteristics of healthy controls and patients with hyperlipidemia.

Variables	Healthy controls (n=72)	Hyperlipidemia (n=75)	P-value
Age (years)	53.24 ± 16.72	55.35 ± 13.91	0.406
Male sex,n (%)	35 (48.61)	37 (48.68)	0.993
BMI (kg/m^2^)	21.77 (19.22–25.41) (n=71)	23.94 (22.43–27.36) (n=71)	0.001
TC (mmol/L)	4.63 (4.07–5.05)	6.04 (5.13–6.62)	<0.001
TG (mmol/L)	0.85 (0.63–1.08)	2.37 (1.41–3.03)	<0.001
LDL-C (mmol/L)	2.69 (2.27–3.05)	3.65 (2.42–4.41)	<0.001
HDL-C (mmol/L)	1.46 (1.30–1.67)	1.09 (0.91–1.35)	<0.001

Data are presented as mean ± standard deviation, n (%), or median (interquartile range), as appropriate. BMI, body mass index; HDL-C, high-density lipoprotein cholesterol; LDL-C, low-density lipoprotein cholesterol; TC, total cholesterol; TG, triglycerides.

While the overall frequency of circulating ILCs did not differ between groups (0.16 [0.12–0.20]% vs. 0.14 [0.10–0.19]%, p=0.153), ILC3s were significantly reduced in hyperlipidemia patients (19.55 ± 8.42 vs. 26.12 ± 10.23, p<0.001) whereas ILC2s were increased (27.94 [15.57–50.35]% vs. 21.56 [11.87–37.06]%, p=0.032; **Figures 2b, c**). To adjust for potential confounding effects on circulating ILC3s, multivariable linear regression analysis was conducted. After adjustment, a lower proportion of ILC3s was still identified in individuals with hyperlipidemia compared with those with normal lipid levels (β=-5.96, SE = 1.59, p<0.001). Similarly, a lower proportion of circulating ILC3s was also noted in overweight individuals (BMI≥25) (β=-3.58, SE = 1.76, p=0.043).

**Figure 3:**
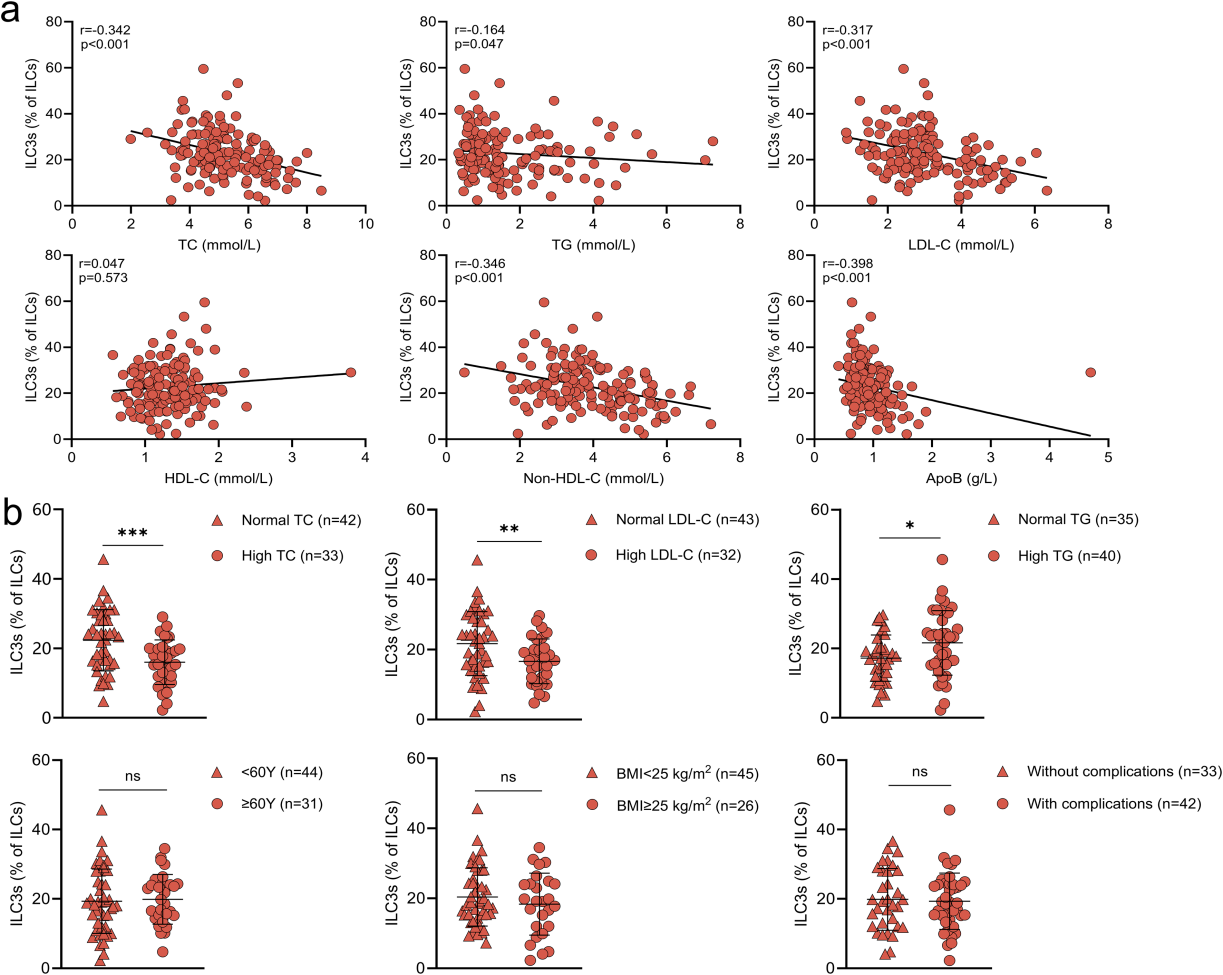
Peripheral ILC3 proportions are inversely correlated with lipid levels **(a)** Correlations between the proportion of peripheral blood ILC3s and lipid parameters, including total cholesterol (TC), triglycerides (TG), low-density lipoprotein cholesterol (LDL-C), high-density lipoprotein cholesterol (HDL-C), non-high-density lipoprotein cholesterol (non-HDL-C), and apolipoprotein B (ApoB). **(b)** Comparison of peripheral ILC3 proportions among hyperlipidemia patients with different clinical characteristics. Data are presented as mean ± standard deviation. *p < 0.05; **p < 0.01; ***p < 0.001; ns, not significant.

Correlation analyses revealed no significant associations between ILC1 or ILC2 frequencies and lipid levels (**Supplementary Figures S3**, **S4**). However, ILC3 frequencies were inversely correlated with TC (r=-0.342, p<0.001), TG (r=-0.164, p=0.047), LDL-C (r=-0.317, p<0.001), non-HDL-C (r=-0.346, p<0.001), and ApoB (r=-0.398, p<0.001) (**Figure 3a**). Subgroup analysis showed that patients with elevated TC or LDL-C had even lower circulating ILC3 proportions (**Figure 3b**).

### 3.3 Integrin α4^+^ ILC3s are a functionally relevant subset associated with hyperlipidemia severity

Integrin α4 is critical for immune cell trafficking to the liver (20), a central organ in lipid metabolism (21). Moreover, integrin α4^+^ ILC3s have been implicated in type 2 diabetes, likely through their regulatory role in glucose metabolism (12). In this study, the proportion of integrin α4^+^ ILC3s in peripheral blood was evaluated.

**Figure 4:**
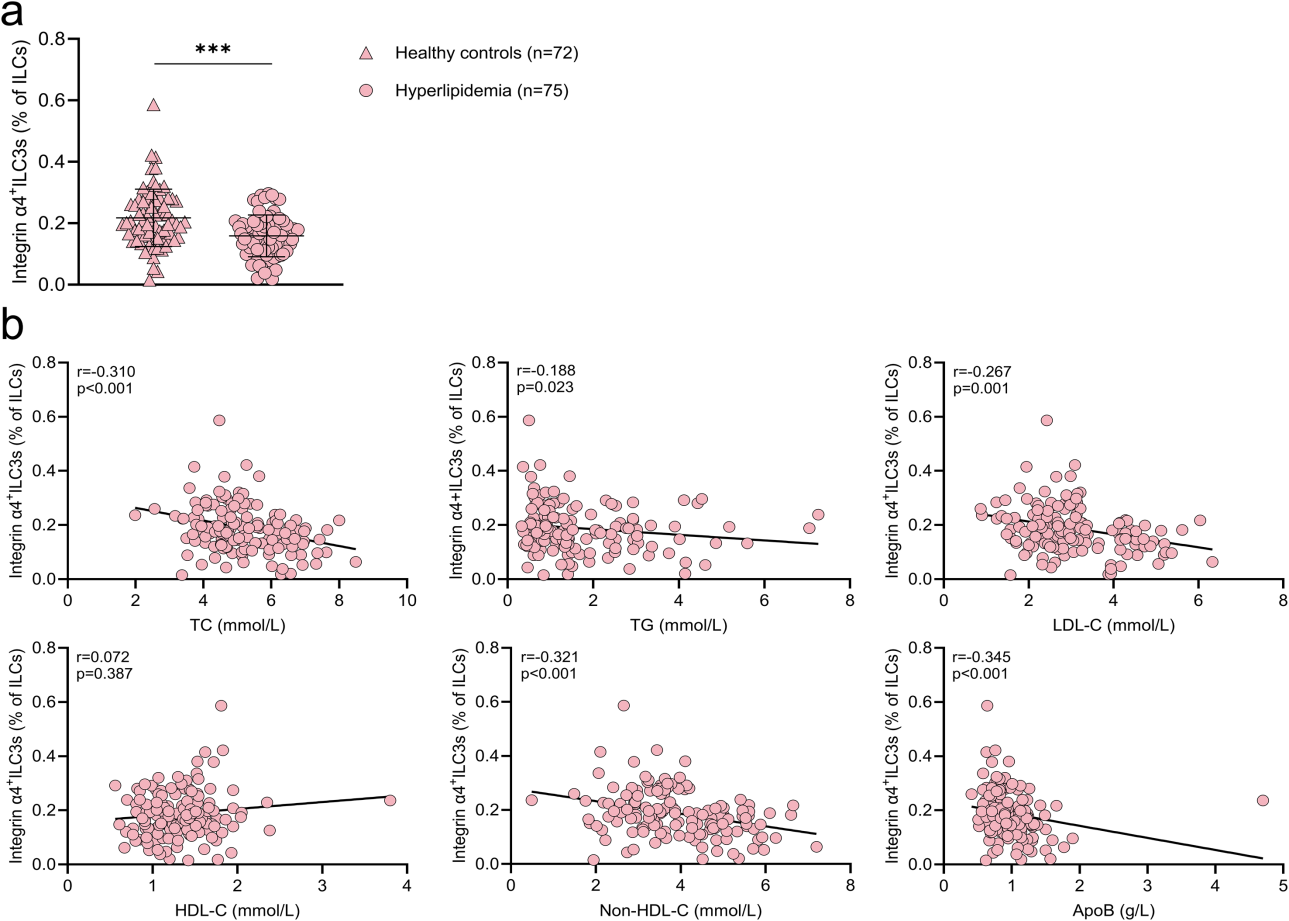
Reduced proportions of peripheral integrin α4^+^ ILC3s in hyperlipidemia and their inverse correlation with disease severity **(a)** Comparison of peripheral integrin α4^+^ ILC3 proportions between healthy controls (n=72) and patients with hyperlipidemia (n=75). **(b)** Correlations between peripheral integrin α4^+^ ILC3 proportions and lipid parameters, including total cholesterol (TC), triglycerides (TG), low-density lipoprotein cholesterol (LDL-C), high-density lipoprotein cholesterol (HDL-C), non-high-density lipoprotein cholesterol (non-HDL-C), and apolipoprotein B (ApoB). ***p < 0.001.

As shown in **Figure 4a**, hyperlipidemia patients exhibited significantly lower proportions of integrin α4^+^ ILC3s compared to healthy controls (0.15 [0.11–0.19]% vs. 0.20 [0.15–0.27]%, p<0.001). These levels negatively correlated with TC (r=-0.310, p<0.001), TG (r=-0.188, p=0.023), LDL-C (r=-0.267, p=0.001), non-HDL-C (r=-0.321, p<0.001), and ApoB (r=-0.345, p<0.001) (**Figure 4b**). Notably, patients with elevated TC showed a more pronounced increase in integrin α4^+^ ILC3s (**Supplementary Figure S5**). These findings indicate that integrin α4^+^ ILC3s may be a lipid-responsive subset involved in disease progression.

### 3.4 ILC3s may regulate lipid metabolism through IL-22 secretion

**Figure 5:**
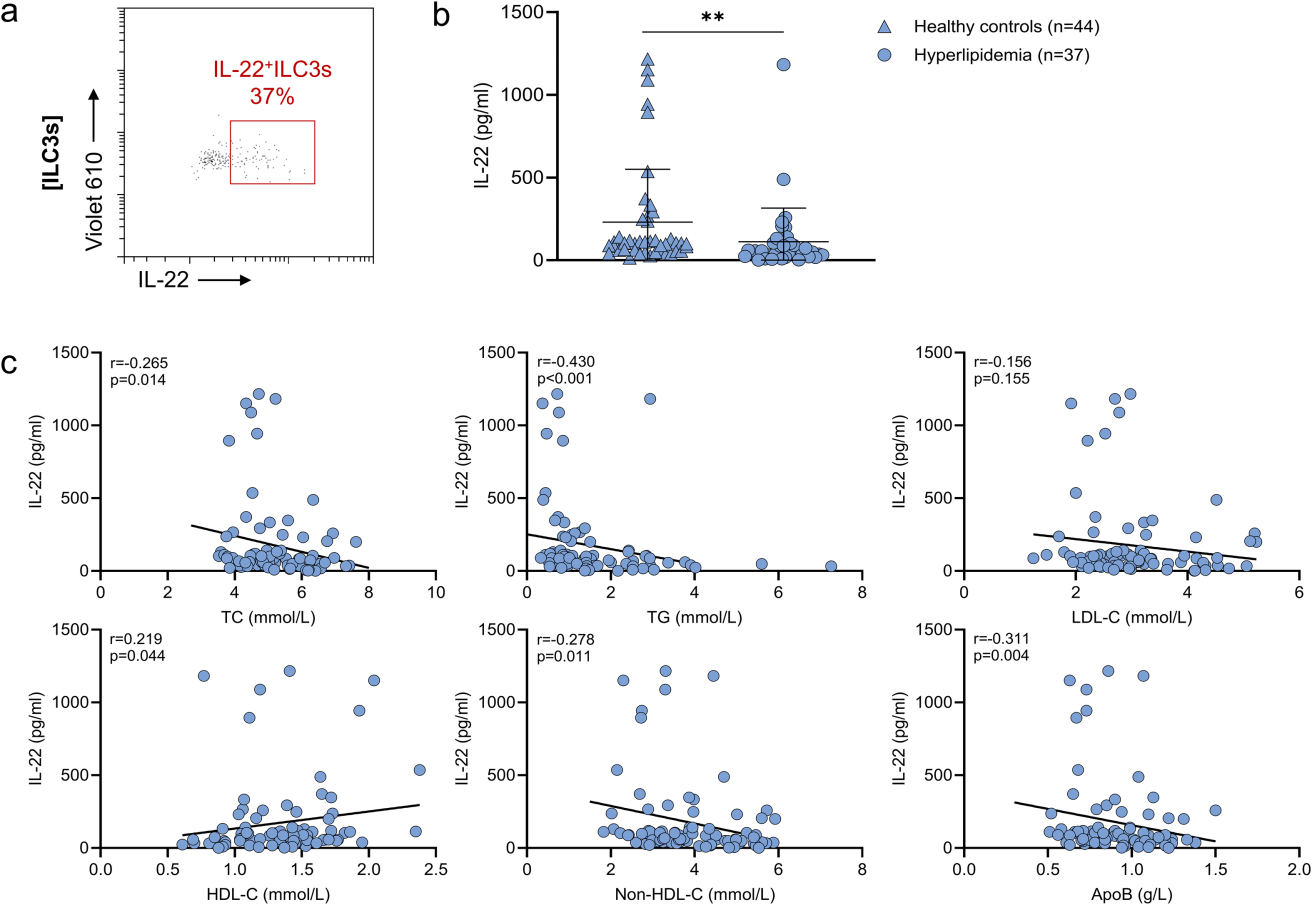
Plasma IL-22 levels are significantly reduced in patients with hyperlipidemia and negatively correlate with lipid parameters **(a)** Representative flow cytometry plot of IL-22^+^ ILC3s in human peripheral blood. **(b)** Comparison of plasma IL-22 levels between healthy controls and patients with hyperlipidemia. **(c)** Correlations between plasma IL-22 levels and lipid parameters, including total cholesterol (TC), triglycerides (TG), low-density lipoprotein cholesterol (LDL-C), high-density lipoprotein cholesterol (HDL-C), non-high-density lipoprotein cholesterol (non-HDL-C), and apolipoprotein B (ApoB). **p < 0.01.

ILC3s are known to produce Th17-like cytokines, particularly IL-17 and IL-22 (22). Among these, IL-22 has been shown to promote hepatic lipid catabolism (11, 16). In addition, flow cytometric analysis revealed that approximately 35% of peripheral blood ILC3s in patients produced IL-22 (**Figure 5a**), indicating that ILC3s represent an important source of this cytokine. Based on these findings, we hypothesized that ILC3s may exert their lipid-lowering effects primarily through IL-22 secretion.

Plasma IL-22 levels were significantly reduced in hyperlipidemia patients compared to healthy controls (49.86 [27.62–103.27] pg/ml vs. 103.95 [57.61–245.27] pg/ml, p=0.001) (**Figure 5b**). Furthermore, IL-22 levels were negatively correlated with TC (r=-0.265, p=0.014), TG (r=-0.430, p<0.001), non-HDL-C (r=-0.278, p=0.011), and ApoB (r=-0.311, p=0.004), and positively correlated with HDL-C (r=0.219, p=0.044) (**Figure 5c**). These results suggest a potential immunometabolic axis wherein reduced IL-22 may contribute to dyslipidemia, and ILC3-derived IL-22 may exert protective metabolic effects.

### 3.5 WMT increases circulating ILC3s and integrin α4^+^ ILC3s, with changes correlating with lipid improvements

**Figure 6:**
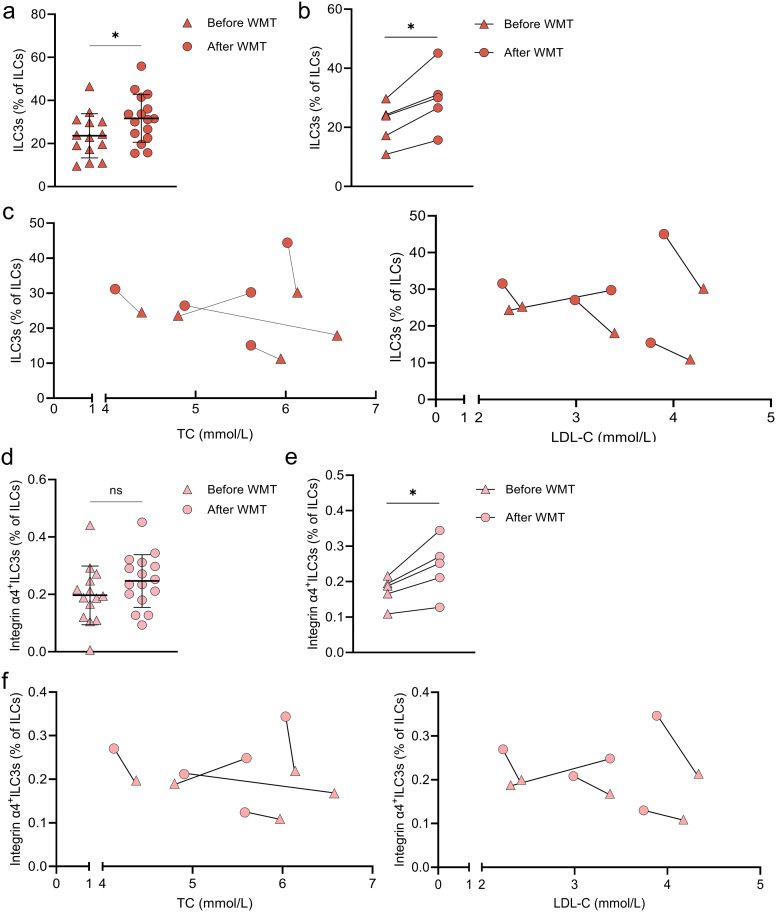
WMT significantly increases peripheral ILC3 and integrin α4^+^ ILC3 proportions in patients with hyperlipidemia **(a)** Proportions of peripheral ILC3s before (n=14) and after (n=16) WMT in unpaired patients with hyperlipidemia. **(b)** Proportions of peripheral ILC3s before and after WMT in paired patients with hyperlipidemia (n=5). **(c)** Correlations between changes in peripheral ILC3 proportions and changes in total cholesterol (TC) and low-density lipoprotein cholesterol (LDL-C) levels. **(d)** Proportions of peripheral integrin α4^+^ ILC3s before (n=14) and after (n=16) WMT in unpaired patients with hyperlipidemia. **(e)** Proportions of peripheral integrin α4^+^ ILC3s before and after WMT in paired patients with hyperlipidemia (n = 5). **(f)** Correlations between changes in peripheral integrin α4^+^ ILC3 proportions and changes in TC and LDL-C levels. *p < 0.05.

Analysis of PBMCs before and after WMT revealed that the proportion of circulating ILC3s was significantly increased following treatment (31.63 ± 11.09% vs. 23.57 ± 10.28%, p=0.049). Further paired analysis of peripheral blood samples from five hyperlipidemia patients confirmed a significant post-WMT increase in circulating ILC3s (29.72 ± 10.55% vs. 21.21 ± 7.28%, p=0.011) (**Figures 6a, b**). Notably, in four of the five patients, the increase in ILC3s was accompanied by reductions in TC and LDL-C (**Figure 6c**).

Similarly, independent sample comparison indicated an upward trend in integrin α4^+^ ILC3s after WMT (0.24 ± 0.09% vs. 0.19 ± 0.10%, p=0.168) (**Figure 6d**). Paired analysis further demonstrated a significant increase in integrin α4^+^ ILC3s following WMT (0.24 ± 0.08% vs. 0.17 ± 0.04%, p=0.022), with four patients showing parallel improvements in lipid profiles (**Figures 6e, f**). These findings suggest that WMT may modulate lipid metabolism by restoring beneficial ILC3 subsets—particularly integrin α4^+^ ILC3s—which may play a key role in systemic lipid regulation.

### 3.6 WMT induces profound shifts in gut microbial composition and plasma metabolites

**Figure 7:**
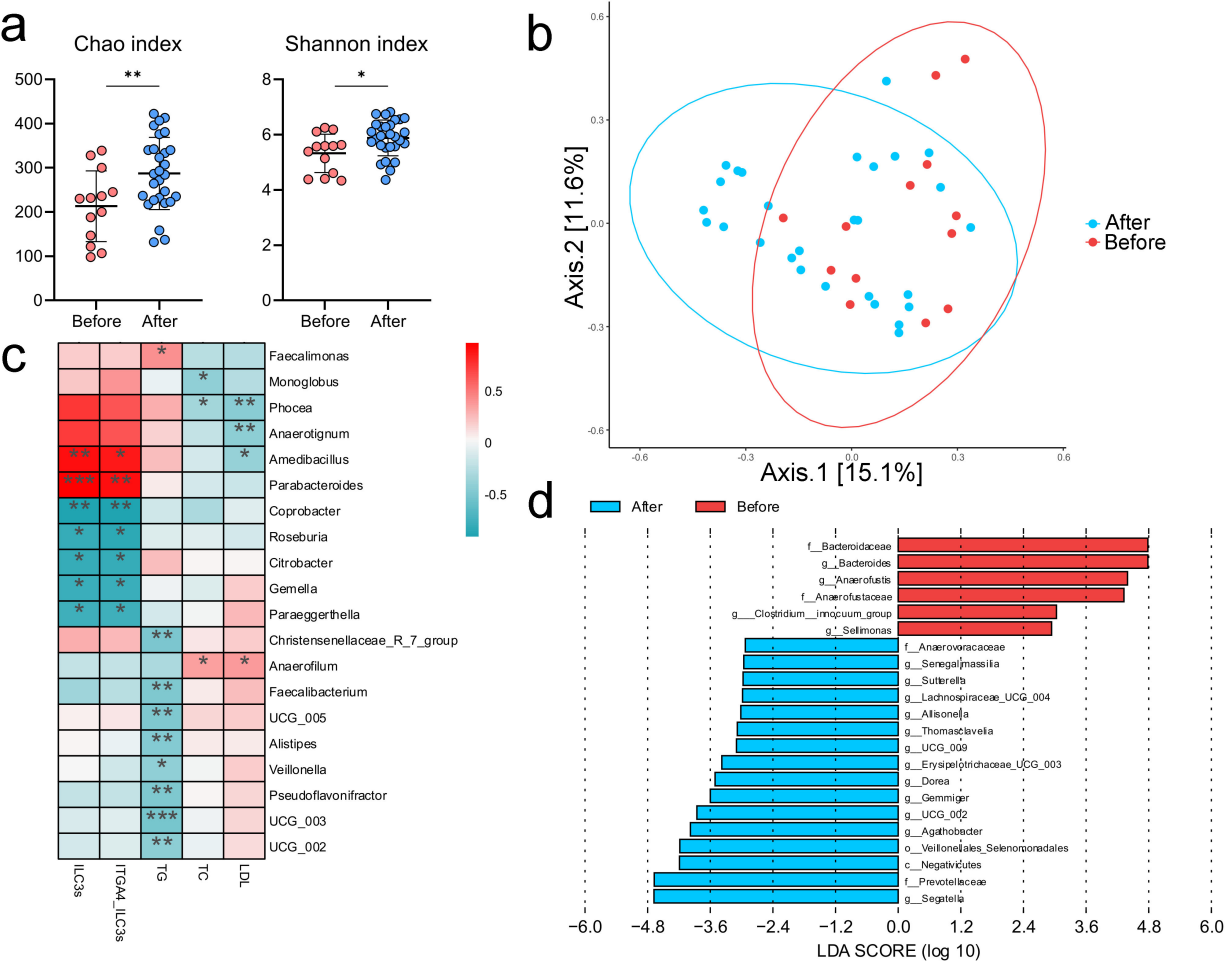
WMT markedly alters the gut microbiota composition in patients with hyperlipidemia **(a)** Differences in the Chao and Shannon indices of the gut microbiota before and after treatment. **(b)** PCoA plots of the gut microbiota before and after treatment. **(c)** Correlations between bacterial genera and patients’ lipid parameters, as well as the proportions of ILC3s and integrin α4^+^ ILC3s. **(d)** Bacterial genera that exhibited significant changes before and after WMT. *p < 0.05; **p < 0.01; ***p < 0.001.

To clarify the impact of WMT on the gut microbiota of patients with hyperlipidemia, 13 pre-treatment and 28 post-treatment fecal samples were subjected to 16S rRNA sequencing. The results showed that both the richness and diversity of the gut microbiota were significantly increased after WMT, accompanied by a distinct shift in microbial community structure (**Figures 7a, b**). In addition, the abundance of *Amedibacillus* was negatively correlated with blood lipid levels but positively correlated with the proportions of ILC3s and integrin α4^+^ ILC3s (**Figure 7c**), suggesting that it may represent a key genus involved in regulating ILC3s and lowering lipid levels. Moreover, the abundance of *UCG-002* was negatively associated with triglycerides, and its levels increased significantly following WMT (**Figures 7c, d**), indicating that WMT may improve hyperlipidemia by modulating *UCG-002*.

**Figure 8:**
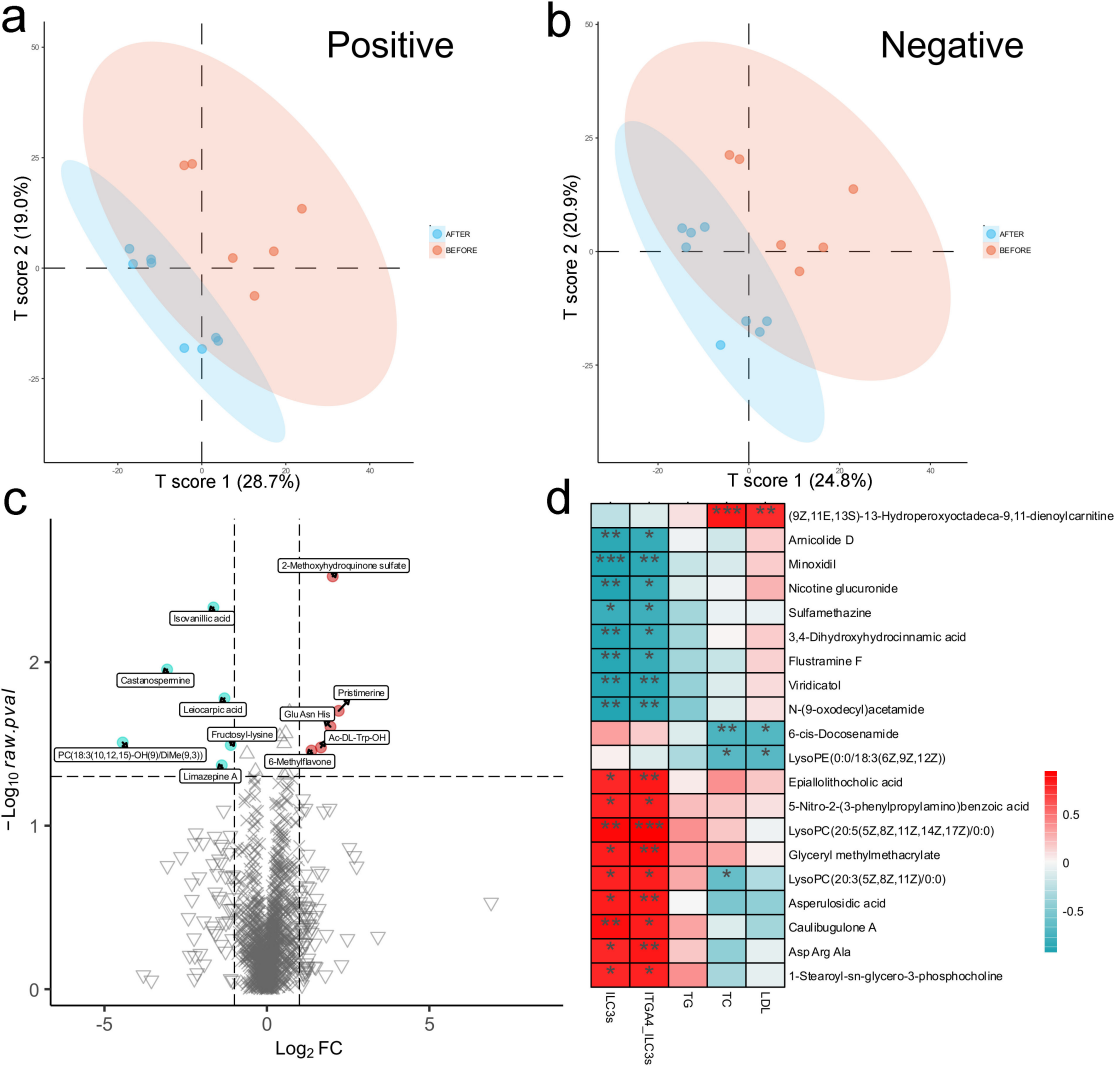
WMT markedly alters the plasma metabolomic profiles of patients with hyperlipidemia. **(a)** OPLS-DA score plot of plasma metabolomics in hyperlipidaemic patients (positive ion mode). **(b)** OPLS-DA score plot of plasma metabolomics in hyperlipidaemic patients (negative ion mode). **(c)** Volcano plot of differential plasma metabolites before and after WMT treatment. **(d)** Correlation analysis between key metabolites and patients’ lipid profiles, as well as the proportions of ILC3s and integrin α4^+^ ILC3s. *p < 0.05; **p < 0.01; ***p < 0.001.

In addition, metabolomic analysis of six pre-treatment and eight post-treatment plasma samples revealed substantial alterations in plasma metabolite profiles following WMT (**Figures 8a, b**). Among these, the abundances of four metabolites—including 2-methoxyhydroquinone sulfate and pristimerine—were significantly increased after WMT, whereas six metabolites—such as isovanillic acid and castanospermine—were markedly decreased (**Figure 8c**). Furthermore, the abundance of lysoPC(20:3(5Z,8Z,11Z)/0:0) showed a positive correlation with the proportions of ILC3s and integrin α4^+^ ILC3s, but a negative correlation with total cholesterol (**Figure 8d**), suggesting that this metabolite may participate in WMT-mediated lipid improvement, potentially through pathways involving ILC3 activation and integrin α4-dependent immune regulation.

### 3.7 Transplantation of a healthy gut microbiota promotes integrin α4^+^ expression in ILC3s and their migration to the liver in hyperlipidemic mice

**Figure 9:**
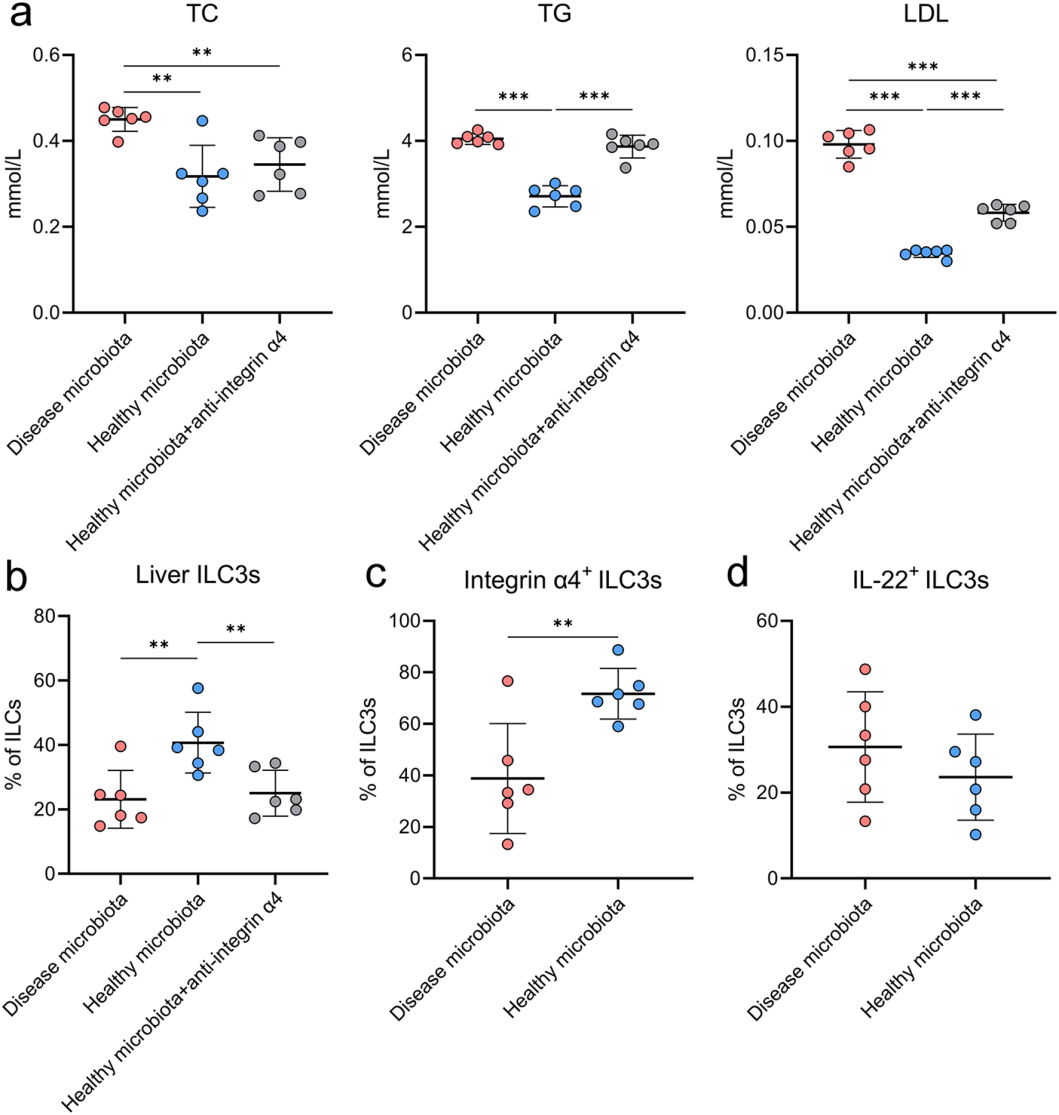
Healthy microbiota transplantation enhances integrin α4 expression on ILC3s, increases hepatic ILC3 abundance, and improves lipid profiles. **(a)** Effects of healthy microbiota transplantation and disease microbiota transplantation on serum lipid levels in hyperlipidemic mice (n=6). **(b)** Effects of healthy microbiota transplantation on the proportion of hepatic ILC3s and the inhibitory impact of integrin α4–neutralizing antibody on ILC3 migration to the liver. **(c)** Effects of healthy microbiota transplantation on the proportion of hepatic integrin α4^+^ ILC3s, and **(d)** on the proportion of hepatic IL-22^+^ ILC3s. **p < 0.01; ***p < 0.001.

In hyperlipidemic mice, transplantation of healthy microbiota significantly reduced serum levels of TC, TG, and LDL (**Figure 9a**), consistent with the clinical findings. Although healthy microbiota transplantation did not increase the proportion of IL-22^+^ ILC3s, it markedly elevated the proportions of hepatic integrin α4^+^ ILC3s and total ILC3s (**Figures 9b-d**), suggesting that healthy microbiota transplantation promotes integrin α4 expression in ILC3s and facilitates their migration to the liver.

To elucidate the role of integrin α4 in ILC3 migration to the liver, hyperlipidemic mice receiving healthy microbiota transplantation were concurrently administered an integrin α4-neutralizing antibody intraperitoneally once weekly for three consecutive weeks. The integrin α4 antibody significantly reduced the hepatic accumulation of ILC3s and attenuated the lipid-lowering effect induced by healthy microbiota transplantation (**Figures 9a, b**), indicating that integrin α4 is a critical molecule mediating the hepatic homing of ILC3s and linking gut microbiota–driven immune cell trafficking to improved lipid metabolism.

Given that hepatic ILC3s have been reported to secrete IL-22 and thereby promote lipid metabolism (11, 16), our findings suggest that a healthy gut microbiota induces integrin α4 expression in ILC3s, facilitates their migration to the liver, and consequently improves lipid metabolism.

## 4 Discussion

This study demonstrates that peripheral ILC3 impairment is a distinct immunological feature of hyperlipidemia. Both total and integrin α4^+^ ILC3s, as well as plasma IL-22, were markedly reduced and showed strong inverse correlations with multiple lipid parameters. WMT administered without lipid-lowering medications improved lipid profiles and simultaneously restored circulating ILC3s and integrin α4^+^ ILC3s, with these immune changes paralleling reductions in TC and LDL-C. WMT also reshaped the gut microbiota and plasma metabolome in a manner associated with ILC3 recovery, and healthy microbiota transplantation in mice increased hepatic ILC3 accumulation through an integrin α4–dependent mechanism (Graphical Abstract). Together, these findings suggest that disruption of the microbiota–ILC3–IL-22 axis contributes to dyslipidemia and that WMT may exert metabolic benefits by restoring this pathway.

Previous studies have demonstrated that WMT improves lipid profiles in patients with hyperlipidemia, even without altering existing lipid-lowering medication regimens (8, 9), and effectively reduces hepatic lipid accumulation in those with fatty liver disease (16). However, the efficacy of WMT as a standalone intervention—absent of any concurrent lipid-lowering therapies—has remained unclear. This study fills that knowledge gap by showing that WMT alone significantly improves lipid profiles in hyperlipidemia patients, with a favorable safety profile. These results highlight the therapeutic potential of gut microbiota modulation in managing dyslipidemia.

Although previous reports have linked ILC3s to lipid metabolism (14, 23, 24), the characteristics of circulating ILC3s in hyperlipidemia have not been well defined. Our findings reveal a significant reduction in peripheral ILC3s among hyperlipidemia patients compared to healthy controls. Notably, this reduction was strongly and inversely correlated with TC, TG, LDL-C, non-HDL-C, and ApoB levels, indicating a potential protective role of ILC3s in systemic lipid homeostasis. This pattern aligns with our previous study in type 2 diabetes, where decreased ILC3 frequencies were similarly associated with greater disease severity (12). Moreover, we observed a marked reduction in integrin α4^+^ ILC3s—a subset implicated in tissue trafficking—which also negatively correlated with key lipid parameters. Given that integrin α4 mediates lymphocyte homing to the liver (21), a central organ in lipid metabolism, the reduction in this subset may reflect impaired gut–liver immune communication, potentially contributing to hepatic lipid dysregulation. Similar reductions in integrin α4^+^ ILC3s in type 2 diabetes further support their role as a sentinel cell population for metabolic disturbances (12).

ILC3s are a major source of IL-22, a cytokine with well-documented roles in mucosal repair, barrier maintenance, and metabolic regulation (25, 26). In our cohort, plasma IL-22 levels were significantly reduced in hyperlipidemia and inversely correlated with TC, TG, non-HDL-C, and ApoB, while positively associated with HDL-C. This cytokine pattern parallels the depletion of ILC3s, suggesting that reduced IL-22 bioavailability may mediate the metabolic consequences of ILC3 deficiency. Animal studies have shown that IL-22 promotes hepatic fatty acid oxidation, suppresses lipogenesis, and protects against steatohepatitis (27, 28). Furthermore, administration of recombinant IL-22 or adoptive transfer of ILC3s reverses diet-induced metabolic disturbances (13). Conversely, high-fat diet exposure has been shown to rapidly impair ILC3s abundance and IL-22 production, leading to intestinal barrier disruption and heightened susceptibility to inflammatory responses (29). Meanwhile, lipid-stress–induced metabolic dysfunction—characterized by reactive oxygen species accumulation and impaired fatty acid oxidation—diminishes ILC3s survival and attenuates IL-22–mediated epithelial repair, establishing a self-reinforcing cycle that progressively exacerbates ILC3s depletion (29, 30). Our human data are consistent with these findings and support a model in which IL-22 acts as a key downstream effector of the ILC3–lipid axis.

Previous studies have shown that FMT can shift the gut microbiota toward a more eubiotic configuration and enhance the production of microbial metabolites, including short-chain fatty acids and tryptophan derivatives, which support ILC3 survival and IL-22 secretion through pathways such as the aryl hydrocarbon receptor (31–33). Our earlier work further demonstrated that WMT promotes the hepatic homing of ILC3s and alleviates liver lipid accumulation in patients with fatty liver disease via IL-22–mediated mechanisms (16). In the present study, marked increases in gut microbial richness and diversity were observed after WMT in patients with hyperlipidemia, accompanied by a significant restructuring of community composition. Notably, *Amedibacillus* displayed negative correlations with lipid parameters and positive correlations with both total and integrin α4^+^ ILC3s, suggesting that this genus may participate in ILC3 regulation and lipid lowering. WMT also significantly increased circulating ILC3s and integrin α4^+^ ILC3s, and these immune changes closely paralleled improvements in TC and LDL-C. In patients with paired immune profiling, increases in ILC3s consistently coincided with reductions in lipid levels, supporting a potential causal link between WMT-induced immune modulation and metabolic improvement despite the limited sample size. In parallel, plasma metabolomics revealed substantial remodeling after WMT, including increased levels of metabolites such as 2-methoxyhydroquinone sulfate and pristimerine, and reductions in metabolites including isovanillic acid and castanospermine. Importantly, lysoPC(20:3) showed positive associations with ILC3 and integrin α4^+^ ILC3 proportions and inverse associations with total cholesterol, indicating that metabolic reprogramming may act in concert with ILC3 restoration to mediate the lipid-lowering effects of WMT.

In addition to the clinical observations, our animal experiments further substantiated the causal role of ILC3s in mediating the lipid-lowering effects of healthy microbiota transplantation. In hyperlipidemic mice, transplantation of a healthy donor microbiota significantly reduced serum triglyceride and cholesterol levels while markedly increasing the hepatic accumulation of ILC3s, particularly the integrin α4–expressing subset, consistent with previous reports showing that integrin α4 is a key molecule regulating lymphocyte homing to the liver through the gut–liver axis (20). These findings align with accumulating evidence that microbial metabolites and microbial structural components can promote ILC3 expansion and IL-22 production, thereby enhancing mucosal and hepatic immune–metabolic homeostasis (16, 34, 35). Importantly, concurrent administration of an integrin α4-neutralizing antibody substantially diminished the hepatic recruitment of ILC3s and attenuated the lipid-lowering efficacy of healthy microbiota transplantation, supporting integrin α4 as a critical mediator of ILC3 trafficking. Collectively, these animal data provide mechanistic evidence that microbiota-induced mobilization of ILC3s—particularly integrin α4^+^ ILC3s—plays a pivotal role in alleviating hepatic lipid accumulation and improving systemic lipid metabolism.

This study has several limitations. First, certain factors that may influence lipid levels—such as dietary habits and exercise frequency during the treatment period—were not documented. Second, the sample size was relatively small and the follow-up duration was short, limiting our ability to assess the persistence of WMT’s lipid-lowering effects, its long-term safety, the durability of post-treatment changes in ILC3s, and the number of treatment courses required to achieve optimal efficacy. Moreover, because this study was not a randomized controlled trial, the potential for bias cannot be excluded. Therefore, the conclusions drawn here require further validation in studies with larger sample sizes and longer follow-up periods.

Overall, our data highlight a microbiota–ILC3 pathway through which washed microbiota transplantation improves lipid metabolism. By promoting the expansion and integrin α4–mediated hepatic homing of ILC3s, WMT contributes to reduced hepatic lipid accumulation in both patients and mice. Although additional mechanistic work is needed, these findings provide a clear foundation for developing microbiota-driven strategies that modulate ILC3 trafficking as a potential therapy for dyslipidemia and metabolic liver disease.

## Data Availability

The datasets presented in this study can be found in online repositories. The names of the repository/repositories and accession number(s) can be found below: https://www.ncbi.nlm.nih.gov/, PRJNA1364992.
