# Supplementary material for: Restoration of peripheral ILC3s by washed microbiota transplantation improves lipid profiles in hyperlipidemia patients
Source: Front Immunol. 2025 Dec 3;16:1688070. doi: 10.3389/fimmu.2025.1688070 (PMC12708897; doi:10.3389/fimmu.2025.1688070)
Supplement: Supplementary file 1 [file Table1.docx]

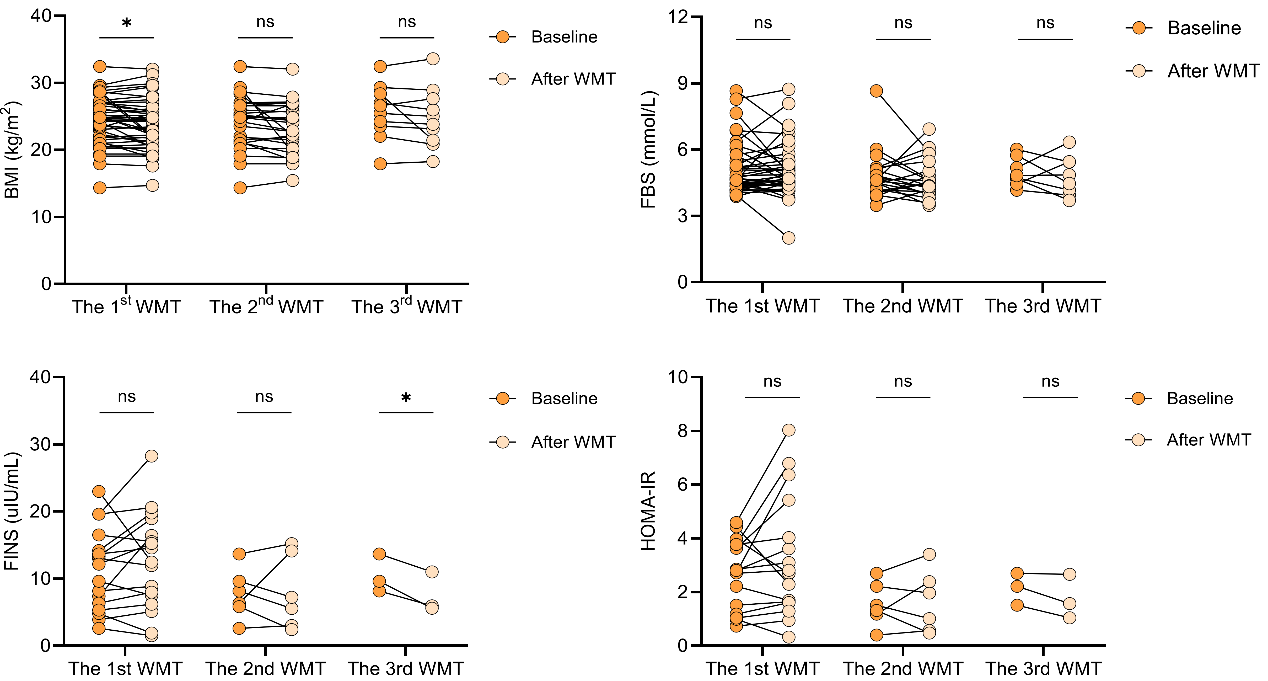


**Figure S1. Effects of WMT on other metabolic parameters in patients with hyperlipidemia**

BMI, body mass index; FBS, fasting blood glucose; FINS, fasting insulin; HOMA-IR, homeostasis model assessment of insulin resistance. *: *p* < 0.05; ns: not significant.


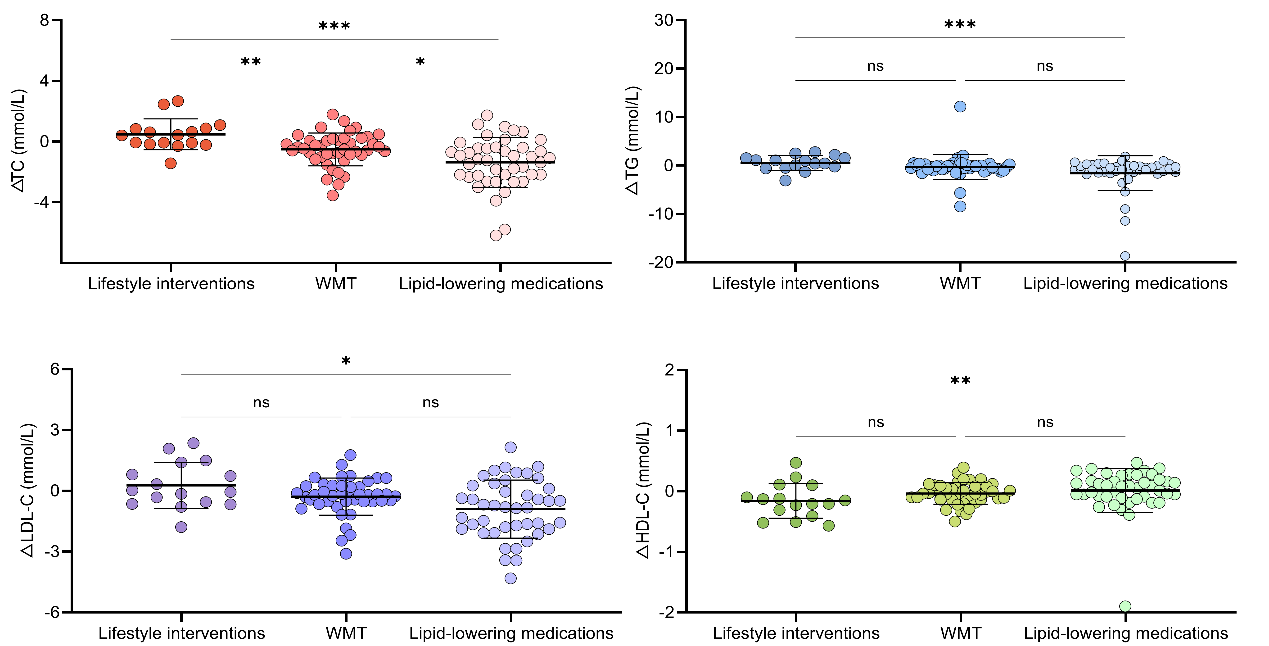


**Figure S2. Effects of lifestyle interventions, WMT, and lipid-lowering medications on patients with hyperlipidaemia**

HDL-C, high-density lipoprotein cholesterol; LDL-C, low-density lipoprotein cholesterol; TC, total cholesterol; TG, triglycerides. *: *p* < 0.05; **: *p* < 0.01; ***: *p* < 0.001.


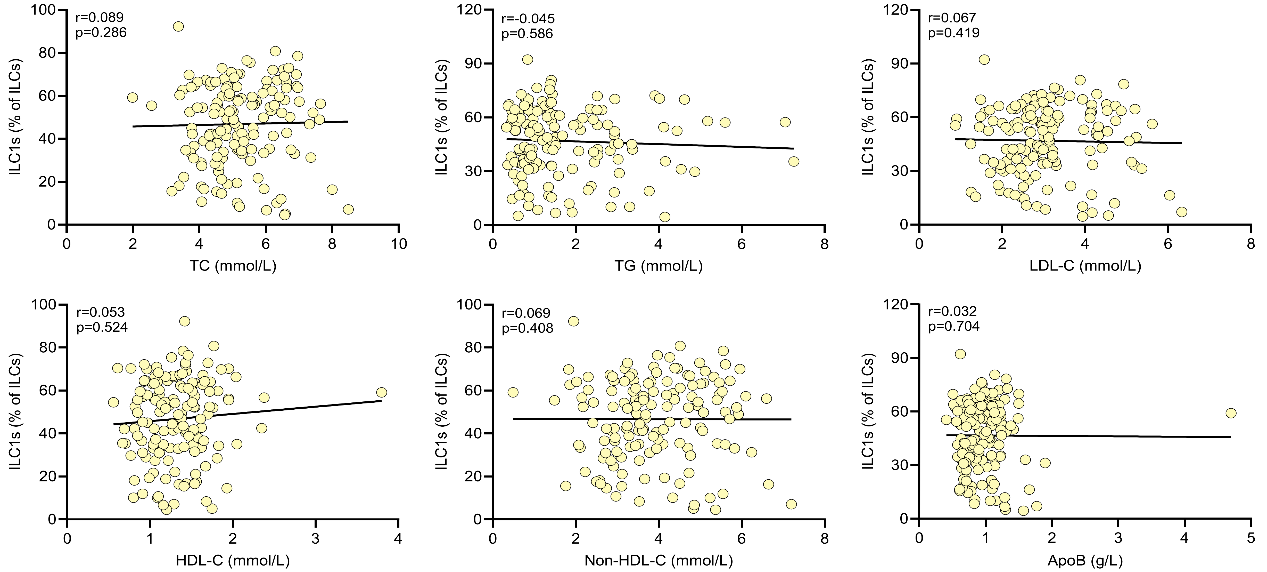


**Figure S3. Correlations between the proportion of peripheral blood ILC1s and lipid parameters**

ApoB, apolipoprotein B; HDL-C, high-density lipoprotein cholesterol; LDL-C, low-density lipoprotein cholesterol; Non-HDL-C, non-high-density lipoprotein cholesterol; TC, total cholesterol; TG, triglycerides.


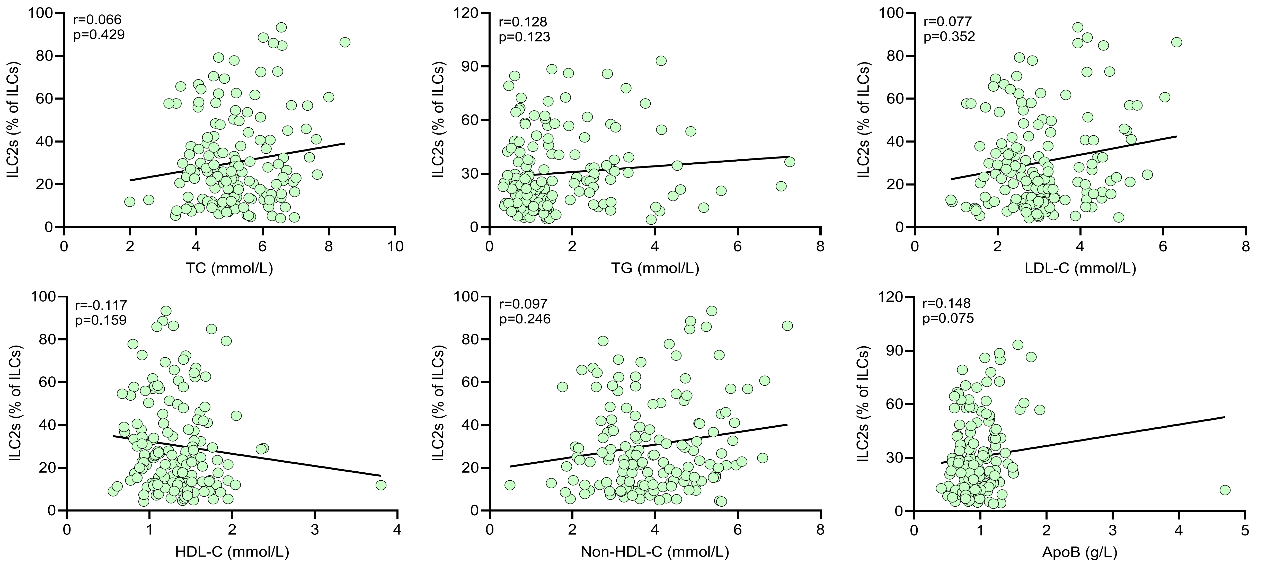


**Figure S4. Correlations between the proportion of peripheral blood ILC2s and lipid parameters**

ApoB, apolipoprotein B; HDL-C, high-density lipoprotein cholesterol; LDL-C, low-density lipoprotein cholesterol; Non-HDL-C, non-high-density lipoprotein cholesterol; TC, total cholesterol; TG, triglycerides.


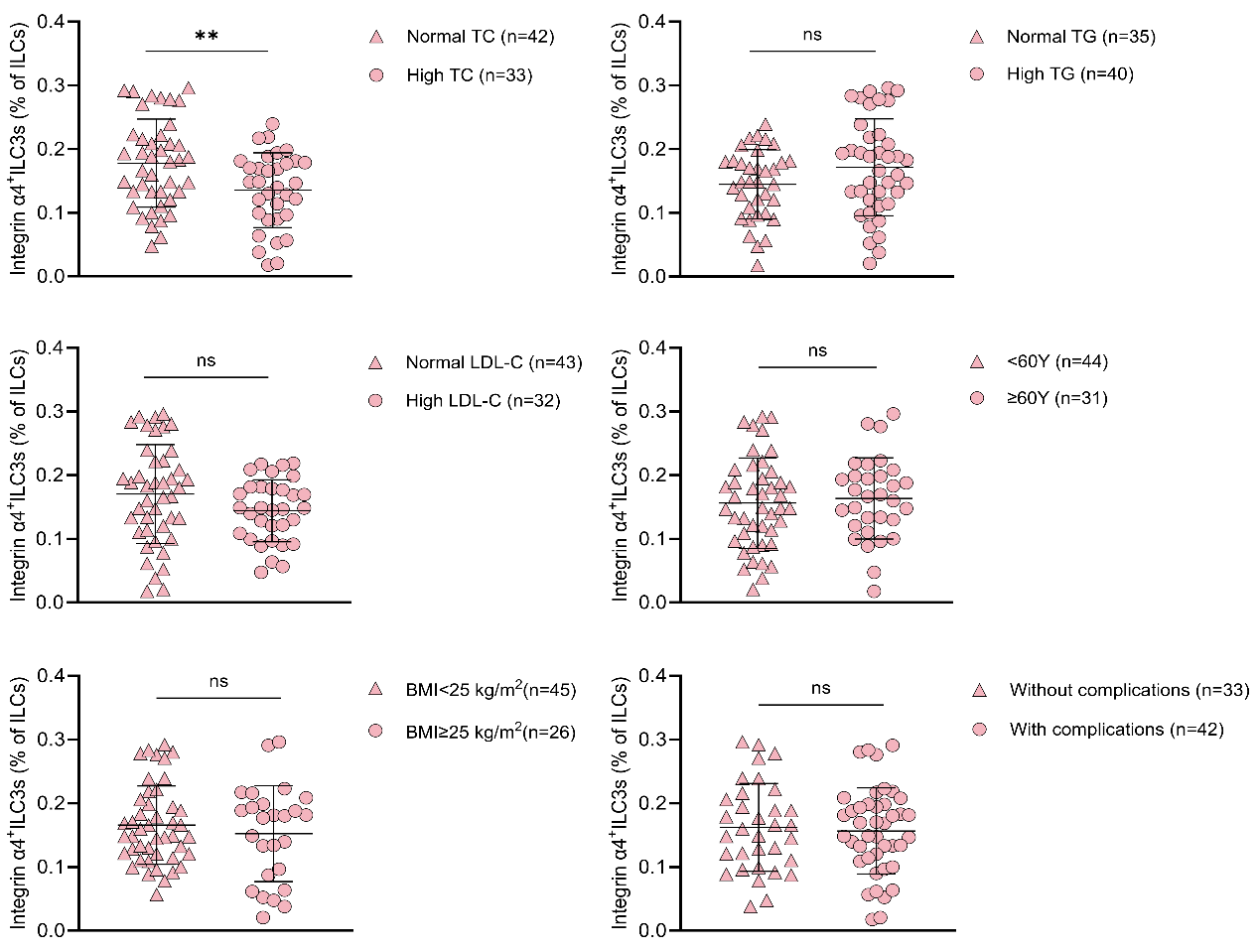


**Figure S5. Comparison of peripheral integrin α4⁺ ILC3 proportions among hyperlipidemia patients with different clinical characteristics**

BMI, body mass index; LDL-C, low-density lipoprotein cholesterol; TC, total cholesterol; TG, triglycerides.

**Table S1. Baseline characteristics of hyperlipidemia patients who underwent washed microbiota transplantation**

|  | Hyperlipidemia (n=46) |
| --- | --- |
| Age (years) | 53.98±12.39 |
| Male sex,n (%) | 25 (54.35) |
| BMI (kg/m^2^) | 24.17±3.73 |
| Smoking status |  |
| Never, n (%) | 40 (86.96) |
| Former, n (%) | 1 (2.17) |
| Current, n (%) | 5 (10.87) |
| Alcoholism, n (%) | 3 (6.52) |
| Hypertension, n (%) | 16 (34.78) |
| Diabetes mellitus type 2, n (%) | 7 (15.22) |

Data are presented as mean ± standard deviation or n (%).

**Table S2. Primary indications for washed microbiota transplantation in patients with hyperlipidemia**

| Primary cause of WMT | Number (n) | Percentage (%) |
| --- | --- | --- |
| Functional bowel disease | 29 | 63.04 |
| Hyperlipidemia | 7 | 15.22 |
| Diabetes mellitus type 2 | 2 | 4.34 |
| Gouty arthritis | 2 | 4.34 |
| Depression | 2 | 4.34 |
| Chemotherapy-associated diarrhea | 1 | 2.17 |
| Chronic hepatitis B | 1 | 2.17 |
| Mediastino-esophageal fistula | 1 | 2.17 |
| Hyperuricemia | 1 | 2.17 |
| Total | 46 | 100 |

Data are presented as n (%).
